# Travel-Associated and Locally Acquired Dengue Cases — United States, 2010–2017

**DOI:** 10.15585/mmwr.mm6906a1

**Published:** 2020-02-14

**Authors:** Aidsa Rivera, Laura E. Adams, Tyler M. Sharp, Jennifer A. Lehman, Stephen H. Waterman, Gabriela Paz-Bailey

**Affiliations:** ^1^Division of Vector-Borne Diseases, National Center for Emerging and Zoonotic Infectious Diseases, CDC, San Juan, Puerto Rico; ^2^Division of Vector-Borne Diseases, National Center for Emerging and Zoonotic Infectious Diseases, CDC, Fort Collins, Colorado.

Dengue is a potentially fatal acute febrile illness caused by any of four mosquito-transmitted dengue viruses (DENV-1 to DENV-4) belonging to the family *Flaviviridae* and endemic throughout the tropics. Competent mosquito vectors of DENV are present in approximately one half of all U.S. counties. To describe epidemiologic trends in travel-associated and locally acquired dengue cases in the United States, CDC analyzed cases reported from the 50 states and District of Columbia to the national arboviral surveillance system (ArboNET). Cases are confirmed by detection of 1) virus RNA by reverse transcription–polymerase chain reaction (RT-PCR) in any body fluid or tissue, 2) DENV antigen in tissue by a validated assay, 3) DENV nonstructural protein 1 (NS1) antigen, or 4) immunoglobulin M (IgM) anti-DENV antibody if the patient did not report travel to an area with other circulating flaviviruses. When travel to an area with other flaviviruses was reported, IgM-positive cases were defined as probable. During 2010–2017, totals of 5,009 (93%) travel-associated and 378 (7%) locally acquired confirmed or probable dengue cases were reported to ArboNET. Cases were equally distributed between males and females, and median age was 41 years. Eighteen (three per 1,000) fatal cases were reported, all among travelers. Travelers should review country-specific recommendations (https://wwwnc.cdc.gov/travel/notices/watch/dengue-asia) for reducing their risk for DENV infection, including using insect repellent and staying in residences with air conditioning or screens on windows and doors.

DENV infection can be asymptomatic or cause disease ranging from a febrile illness with headache, myalgia, arthralgia, and rash, to potentially fatal manifestation of severe dengue, including plasma leakage, hemorrhage, or severe organ impairment. The four DENVs are endemic throughout the tropics and are common causes of acute febrile illness in travelers (*1*). Globally, the number of dengue cases doubled each decade from 1990 to 2013, reaching an estimated maximum of 390 million (95% credible interval [CI]* = 284–528) DENV infections in 2010, 96 million (95% CI = 67–136) of which resulted in symptomatic cases (*2*). An estimated average of 13,600 (95% uncertainty interval [UI]^†^ = 4,200–34,700) persons die from dengue every year (*3*). The geographic range of dengue is expected to further expand as a result of rising world temperatures and urbanization (*4*). Infection with a DENV produces long-lasting immunity to that virus; however, persons later infected with another DENV can be at increased risk for developing severe dengue (*5*).

Although multiple behavioral, environmental, and entomologic approaches have been implemented to control *Aedes* spp. mosquito populations, none has yet proven to be both sustainable and effective. No specific treatment for dengue exists. The first Food and Drug Administration–approved vaccine against dengue, Dengvaxia, is licensed for use in approximately 20 countries, and was recently approved for use in the United States in children aged 9–16 years who have laboratory evidence of prior DENV infection and who live in areas with endemic DENV (*6*). The Advisory Committee on Immunization Practices has not yet issued recommendations for Dengvaxia use in the United States. Other candidate vaccines are undergoing clinical trials.

In 2010, dengue became a nationally notifiable disease; state and territorial health departments report dengue cases to CDC through ArboNET (https://www.cdc.gov/dengue/statistics-maps/index.html; https://wwwn.cdc.gov/nndss/conditions/dengue/). This report describes locally acquired and travel-associated, laboratory-confirmed and probable dengue cases reported to ArboNET from the 50 states and District of Columbia with illness onset during January 1, 2010–December 31, 2017.

Dengue cases were described according to the Council of State and Territorial Epidemiologists case definitions (*7*). Confirmed cases met the clinical criteria and had detection of 1) DENV nucleic acid by RT-PCR in any body fluid or tissue, 2) DENV antigen in tissue by a validated assay, 3) DENV NS1 antigen by a validated immunoassay, or 4) IgM anti-DENV antibody if exposure occurred in an area without evidence of other flavivirus transmission. Probable dengue cases met the clinical criteria and were defined by detection of IgM anti-DENV antibody in serum if the person lived in or traveled to an area with transmission of another flavivirus. The infecting DENV was determined by molecular typing by RT-PCR (https://www.cdc.gov/dengue/healthcare-providers/testing/molecular-tests/index.html). Travel destinations were recorded as the areas visited outside the continental United States in the 14 days before illness onset, considered as the most likely locations of infection. The incidence of dengue cases among U.S. outbound travelers was calculated using denominator data from the National Travel and Tourism Office.^§^ Travelers to Europe were excluded from the denominator because of the small number of dengue cases reported from Europe.

During 2010–2017, a total of 5,387 dengue cases were reported to ArboNET, 5,009 (93%) of which were travel-associated; 378 (7%) were locally acquired (Table 1). Two thirds were probable cases. Cases were equally distributed between males and females, and the median patient age was 41 years. Nearly half (46%) of patients were white, and 14% were Asian. Among 459 cases for which the infecting DENV was identified, DENV-1 (308 cases, 67%) was the most common (Table 2). The largest number of dengue cases (961, 18%) was reported in 2016, and the smallest (254, 5%) in 2011. The average annual number of travel-associated dengue cases was 626, and the average annual incidence was 16 cases per 1 million U.S. travelers (range = 7–28).

**TABLE 1:** Reported number of travel-associated dengue cases, by state — United States, 2010–2017

State	Travel-associated cases No. (%)
Alabama	28 (1)
Alaska	14 (0)
Arizona	153 (3)
Arkansas	11 (0)
California	819 (16)
Colorado	49 (1)
Connecticut	55 (1)
Delaware	9 (0)
District of Columbia	27 (1)
Florida*	695 (14)
Georgia	75 (1)
Hawaii*	86 (2)
Idaho	13 (0)
Illinois	172 (3)
Indiana	48 (1)
Iowa	35 (1)
Kansas	25 (0)
Kentucky	11 (0)
Louisiana	33 (1)
Maine	14 (0)
Maryland	69 (1)
Massachusetts	38 (1)
Michigan	87 (2)
Minnesota	115 (2)
Mississippi	8 (0)
Missouri	36 (1)
Montana	19 (0)
Nebraska	13 (0)
Nevada	21 (0)
New Hampshire	13 (0)
New Jersey	249 (5)
New Mexico	11 (0)
New York*	878 (18)
North Carolina	65 (1)
North Dakota	6 (0)
Ohio	65 (1)
Oklahoma	19 (0)
Oregon	21 (0)
Pennsylvania	137 (3)
Rhode Island	24 (0)
South Carolina	40 (1)
South Dakota	10 (0)
Tennessee	47 (1)
Texas*	267 (5)
Utah	14 (0)
Vermont	19 (0)
Virginia	140 (3)
Washington	127 (3)
West Virginia	6 (0)
Wisconsin	72 (1)
Wyoming	1 (0)
**Total**	**5,009**

* In addition to travel-associated cases, these four states reported a total of 378 locally acquired cases: Hawaii (250 cases), Florida (103), Texas (24), and New York (one).

**TABLE 2:** Characteristics of reported travel-associated and locally acquired dengue cases — ArboNET, United States, 2010–2017

Characteristic	No. (%)		
	Travel-associated cases (n = 5,009)	Locally acquired cases (n = 378)	Total (N = 5,387)
**Case definition**			
Probable	3,539 (71)	58 (15)	3,597 (67)
Confirmed	1,470 (29)	320 (85)	1,790 (33)
Infecting DENV*			
DENV-1	119 (45)	189 (96)	308 (67)
DENV-2	71 (27)	4 (2)	75 (16)
DENV-3	45 (17)	3 (2)	48 (10)
DENV-4	28 (11)	0 (0)	28 (6)
**Sex^†^**			
Female	2,500 (50)	188 (50)	2,688 (50)
Male	2,508 (50)	190 (50)	2,698 (50)
**Race**			
White	2,240 (45)	245 (65)	2,485 (46)
Asian	729 (15)	24 (6)	753 (14)
Black or African American	277 (6)	5 (1)	282 (5)
Native Hawaiian or other Pacific Islander	37 (1)	72 (19)	109 (2)
American Indian or Alaska Native	16 (0)	5 (1)	21 (0)
Asian, White	2 (0)	0 (0)	2 (0)
Asian, Native Hawaiian or other Pacific Islander	0 (0)	1 (0)	1 (0)
Unknown	1,708 (34)	26 (7)	1,734 (32)
**Age group (yrs)^§^**			
0–9	129 (3)	14 (4)	143 (3)
10–19	515 (10)	46 (12)	561 (10)
20–29	887 (18)	67 (18)	954 (18)
30–39	847 (17)	43 (11)	890 (17)
40–49	897 (18)	70 (19)	967 (18)
50–59	904 (18)	57 (15)	961 (18)
60–69	574 (11)	56 (15)	630 (12)
≥70	246 (5)	24 (6)	270 (5)
**Region of travel**			
Caribbean	1,649 (33)	—	1,649 (33)
Asia	1,469 (29)	—	1,469 (29)
Central America	676 (14)	—	676 (14)
North America^¶^	477 (10)	—	477 (10)
South America	327 (7)	—	327 (7)
Unknown	222 (4)	—	222 (4)
Africa	89 (2)	—	89 (2)
Oceania	85 (2)	—	85 (2)
Europe	7 (<1)	—	7 (<1)
Multiple regions	8 (<1)	—	8 (<1)
Clinical syndrome**			
Dengue^††^	4,597 (94)	353 (94)	4,950 (94)
Dengue-like illness^§§^	254 (5)	24 (6)	278 (5)
Severe dengue^¶¶^	46 (<1)	1 (<1)	47 (<1)
**Outcome**			
Hospitalized	2,119 (42)	57 (15)	2,176 (40)
Died	18 (<1)	0	18 (<1)

* Not available before 2014 (n = 459).

^†^ One unknown sex among travelers.

^§^ Ten unknown age group among travelers and one among locally acquired cases.

^¶^ 99% of patients (472) traveled to Mexico.

** National Notifiable Diseases Surveillance System (NNDSS) dengue definitions from 2010 and 2015. Dengue hemorrhagic fever and dengue shock syndrome cases were classified as severe dengue in this analysis; dengue fever and dengue fever with hemorrhage cases were classified as dengue. https://wwwn.cdc.gov/nndss/conditions/dengue-virus-infections/case-definition/2015/.

^††^ Dengue is defined by fever as reported by the patient or health care provider and the presence of one or more of the following signs and symptoms: nausea/vomiting, rash, aches and pains (e.g., headache, retro-orbital pain, joint pain, myalgia, or arthralgia), tourniquet test positive, leukopenia (a total white blood cell count of <5,000/mm^3^), or any warning sign for severe dengue: abdominal pain or tenderness, persistent vomiting, extravascular fluid accumulation (e.g., pleural or pericardial effusion or ascites), mucosal bleeding at any site, liver enlargement >2 cm, or increasing hematocrit concurrent with rapid decrease in platelet count.

^§§^ Dengue-like illness (59 cases) was combined with febrile illness (two), and uncomplicated fever (217); 91 cases with unknown clinical syndrome and 21 classified as other clinical syndrome were excluded, all of them were travel-associated.

^¶¶^ Severe dengue is defined as dengue with any one or more of the following: 1) severe plasma leakage evidenced by hypovolemic shock or extravascular fluid accumulation (e.g., pleural or pericardial effusion or ascites) with respiratory distress; 2) severe bleeding from the gastrointestinal tract (e.g., hematemesis or melena) or vagina (menorrhagia) as defined by requirement for medical intervention including intravenous fluid resuscitation or blood transfusion, or 3) severe organ involvement, including any of the following: elevated liver transaminases: aspartate aminotransferase or alanine aminotransferase ≥1,000 per liter (U/L), impaired level of consciousness or diagnosis of encephalitis, encephalopathy, or meningitis, or heart or other organ involvement including myocarditis, cholecystitis, and pancreatitis.

Approximately one half (53%) of travel-associated cases were reported from four states: New York (18%), California (16%), Florida (14%), and Texas (5%) (Table 1). Travel history was reported for 96% of cases. The most frequently reported regions of travel were the Caribbean (33%) and Asia (29%), followed by Central America (14%), North America (10%) and South America (7%) (Table 2). The most frequently reported region of travel changed from the Caribbean (42%) during 2010–2014 to Asia (35%) during 2015–2017 (Figure). The most frequently reported destinations with endemic transmission across all years were the countries of India (591, 12%), Mexico (472, 9%), and Dominican Republic (443, 9%), and the U.S. territory of Puerto Rico (343, 7%).

**FIGURE:**
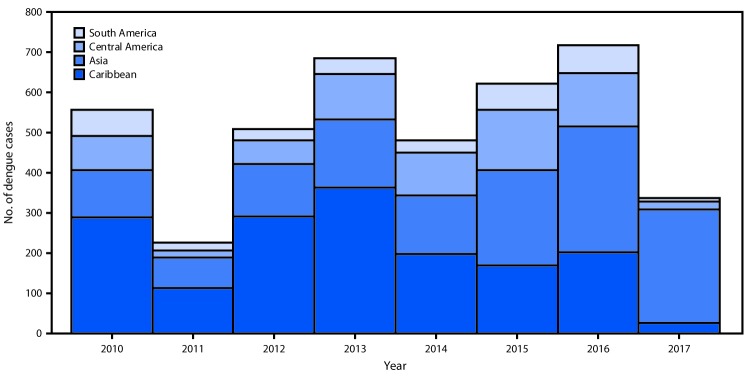
Number of travel-associated dengue cases in U.S. residents, by reported travel destination and year of illness onset — 2010–2017

Hawaii reported the largest number of locally acquired dengue cases (250; 66%), followed by Florida (103; 27%), Texas (24; 6%), and New York (one; 0.3%) (Table 1). All locally acquired cases in Hawaii (98%) were reported during a 2015–2016 outbreak, whereas most cases in Florida were reported during outbreaks in Monroe County in 2010 (56 cases) and in Martin County in 2013 (17). Texas reported a small outbreak in 2013 with most cases (21) in Cameron County.

The majority of patients with travel-associated (94%) and locally acquired (94%) dengue had reported symptoms consistent with dengue; a small percentage of patients with travel-associated (<1%) and locally acquired (<1%) cases had severe dengue. Overall, 2,176 (40%) patients with dengue were hospitalized, most of whom (2,119; 97%) were travelers. Eighteen (three per 1,000) fatal dengue cases were reported, all of which occurred in travelers (Table 2). The median age of patients with fatal dengue was 47 years (range 21–80 years). Region of birth was available for two of the decedents (one each from the Pacific and Central American regions).

## Discussion

Most dengue cases reported in the 50 states and District of Columbia during 2010–2017 were in adults and were associated with travel to the Caribbean and Asia. Travel-associated cases were reported primarily from New York, California, Florida, and Texas. The most common travel destinations shifted over time, underscoring the importance of travelers being vigilant and reviewing current dengue trends before travel (https://wwwnc.cdc.gov/travel). Locally acquired cases occurred in four states, three of which (Florida, Hawaii, and Texas) also experienced local outbreaks. These data, especially the comparatively large outbreak in Hawaii, demonstrate the ongoing risk for local DENV transmission in *Aedes*-infested areas of the United States following introduction by travelers returning from the tropics.

Competent mosquito vectors of DENV are present in approximately half of all U.S. counties, and an estimated 71% of counties are environmentally suitable for *Aedes aegypti*, the most efficient DENV vector (*8*). Recent dengue outbreaks in the United States have been limited, likely because of lifestyle differences, including the use of screens in U.S. homes and air conditioning that limit exposure to mosquitoes (*9*). However, the trend toward more frequent travel of U.S. residents to the tropics increases the possibility of local dengue outbreaks, including in jurisdictions where local cases have not occurred in recent years (*4*). The number of travel-associated dengue cases peaked at approximately 900 in 2016 and could increase if large dengue epidemics occur in the Region of the Americas. Dengue surveillance is a critical public health task because of the presence of *Aedes aegypti* in many jurisdictions and the risk for virus introduction. Although dengue incidence in travelers is low, health agencies must remain vigilant because most cases are asymptomatic and reported cases represent a small percentage of all infections.

The findings in this report are subject to at least three limitations. First, reporting of dengue symptoms was incomplete. Second, the clinical features of dengue are similar to those for other acute febrile illnesses, including chikungunya and Zika virus disease, which complicates identification, diagnostic testing, and reporting of dengue patients and likely results in an underestimate of the true incidence of travel-associated and locally acquired dengue cases. In addition, the case definition was modified in 2015 to classify dengue hemorrhagic fever and dengue shock syndrome as severe dengue and dengue fever and dengue fever with hemorrhage as dengue (*7*); thus, annual trends might not be comparable.

Dengue is endemic in South and Central America, the Caribbean, Southeast Asia, and central Africa, and more than half of the global population live in areas that are suitable for DENV transmission (*4*). Travelers to and residents of areas with risk for DENV infection should implement personal protection measures to avoid mosquito bites, including using insect repellent, wearing long pants and long sleeves, and staying in residences with air conditioning or screened windows and doors.^¶^ When conducting pretravel consultations, clinicians should include discussion of dengue risk, mosquito avoidance strategies, and advice about seeking health care for febrile illnesses occurring during or after travel. Clinicians should consider dengue when evaluating patients with acute febrile illness and recent travel to the tropics and should consider recommended diagnostic testing (*10*). Suspected dengue cases should be reported to public health authorities to enable timely responses.

**Summary**

**What is already known about this topic?**

The four dengue viruses are transmitted by *Aedes* spp. mosquitoes and are common causes of acute febrile illness in travelers visiting the tropics.

**What is added by this report?**

During 2010–2017, a total of 5,387 dengue cases were reported from U.S. states; 93% were travel-associated. Locally acquired cases were reported from Hawaii (250 cases), Florida (103), Texas (24), and New York (one).

**What are the implications for public health practice?**

Travelers to the tropics should protect against mosquito bites by using insect repellents, wearing long-sleeved shirts and long pants, and taking actions to keep mosquitos out of their residences. Clinicians should remain vigilant for and report suspected dengue cases to local health authorities.

## Footnotes

* Differs from a confidence interval; a credible interval is the interval in which an unobserved parameter has a given probability, dependent on the prior distribution.

† Another term for confidence interval.

§
https://travel.trade.gov/research/monthly/departures/ .

¶
https://wwwnc.cdc.gov/travel/diseases/dengue .
